# Nosocomial transmission clusters and lineage diversity characterized by SARS-CoV-2 genomes from two large hospitals in Paris, France, in 2020

**DOI:** 10.1038/s41598-022-05085-2

**Published:** 2022-01-20

**Authors:** Valentin Leducq, Aude Jary, Antoine Bridier-Nahmias, Lena Daniel, Karen Zafilaza, Florence Damond, Valérie Goldstein, Audrey Duval, François Blanquart, Vincent Calvez, Diane Descamps, Anne-Geneviève Marcelin, Benoit Visseaux

**Affiliations:** 1 Sorbonne Université, INSERM, Institut Pierre Louis d’Epidémiologie et de Santé Publique (iPLESP), AP-HP, Hôpital Pitié-Salpêtrière, Service de Virologie, 47-83 Bd de l’hôpital, 75013 Paris, France; 2 Université de Paris, Inserm, UMR1137, IAME, Paris, France; 3 Université de Paris, Inserm, UMR1137, IAME, Service de Virologie, Hôpital Bichat-Claude Bernard, AP-HP, Paris, France; 4 AP-HP, Sorbonne Université, Hôpital Pitié-Salpêtrière Charles-Foix, Service de Bactériologie Hygiène, Paris, France; 5 Centre for Interdisciplinary Research in Biology (CIRB), Collège de France, CNRS, INSERM, PSL Research University, Paris, France

**Keywords:** SARS-CoV-2, Viral epidemiology, Viral evolution, Epidemiology, Next-generation sequencing, Viral infection, Phylogeny

## Abstract

France went through three deadly epidemic waves due to the severe acute respiratory syndrome coronavirus 2 (SARS-CoV-2), causing major public health and socioeconomic issues. We proposed to study the course of the pandemic along 2020 from the outlook of two major Parisian hospitals earliest involved in the fight against COVID-19. Genome sequencing and phylogenetic analysis were performed on samples from patients and health care workers (HCWs) from Bichat (BCB) and Pitié-Salpêtrière (PSL) hospitals. A tree-based phylogenetic clustering method and epidemiological data were used to investigate suspected nosocomial transmission clusters. Clades 20A, 20B and 20C were prevalent during the spring wave and, following summer, clades 20A.EU2 and 20E.EU1 emerged and took over. Phylogenetic clustering identified 57 potential transmission clusters. Epidemiological connections between participants were found for 17 of these, with a higher proportion of HCWs. The joint presence of HCWs and patients suggest viral contaminations between these two groups. We provide an enhanced overview of SARS-CoV-2 phylogenetic changes over 2020 in the Paris area, one of the regions with highest incidence in France. Despite the low genetic diversity displayed by the SARS-CoV-2, we showed that phylogenetic analysis, along with comprehensive epidemiological data, helps to identify and investigate healthcare associated clusters.

## Introduction

In December 2019, an increasing number of severe respiratory infections of unknown etiology have been reported in the province of Hubei, China, leading to the identification of a novel human coronavirus, the Severe Acute Respiratory Syndrome CoronaVirus 2 (SARS-CoV-2), responsible for the COronaVIrus Disease 2019 (COVID-19)^1^. Within a few months, the SARS-CoV-2 has been spreading worldwide and the World Health Organization (WHO) declared the COVID-19 outbreak a global pandemic on the 11th of March 2020. The pandemic has been responsible for more than 4 million of deaths and major public health and socioeconomic issues across the globe^2^. The effort of the scientific community helped understand the epidemiology of COVID-19 and guide public health policies to provide an efficient response. Notably, viral genome sequencing capacities have increased dramatically, enabling for a close monitoring of the epidemic’s dynamic and the evolution of the SARS-CoV-2 genome. Databases and tools such as GISAID (https://www.gisaid.org/) and Nextstrain (https://clades.nextstrain.org/), gathering and providing real time analysis of the SARS-CoV-2 sequences’ diversity, helped monitor the global spread of the epidemic.

Mutation rates of RNA viruses are extremely high—up to a million times higher than their hosts—and allow great evolving and adapting abilities^3^. Although SARS-CoV-2 evolves more slowly than other RNA viruses^4^, a variant harboring the Spike amino acid change D614G has been spotted in Germany in late January, as the epidemic reached Europe^5^. This variant (nextstrain clade 20A) caused large outbreaks in early 2020 and became the most prevalent lineage by April in most European countries and worldwide, suggesting a possible increased infectivity^6^. Subsequently, the 20A clade gave rise to the 20B and 20C clades harboring additional mutations (G28881A/G28882A/G28883C and C1059T/G25563T, respectively). Together, these three clades constituted most of the SARS-CoV-2 diversity during the spring COVID-19 wave in Europe. In summer, despite low viral circulation, two novel 20A related variants with additional mutations emerged. The 20E.EU1 (Pango lineage B.1.177) associated with Spike A222V mutation, first spotted in Spain^7^, and the 20A.EU2 (Pango lineage B.1.160) associated with Spike S477N mutation with earliest sequences identified in France^8^. Along with lockdown suspensions and borders reopening, 20E.EU1 and 20A.EU2 spread across Europe and seeded new outbreaks^9^. These two latest clades quickly became dominant in most European countries during the fall COVID-19 wave.

In France the first wave struck as a sudden spring outbreak, from March to April 2020, followed by a drop in the number of contaminations in response to the nationwide lockdown. In late summer, an increase of reported contaminations was observed and led to a second epidemic wave in fall, requiring a second nationwide lockdown in November. France remains one of the most affected countries by the COVID-19, with more than 5.6 million confirmed cases and more than 110.000 deaths in July 2021. Due to its high population density and a large mixing of populations from various regions and countries, the Paris urban area was one of the regions with the highest number of COVID-19 cases and incidence levels in 2020. Nevertheless, only 676 full SARS-CoV-2 genomes were available so far to describe the molecular diversity and the evolution of SARS-CoV-2 within this major European megalopolis. Bichat Claude-Bernard and Pitié-Salpêtrière hospitals were the two first hospitals of the Paris urban area operational for the diagnosis and care of patients with SARS-CoV-2 infections. From the onset of COVID-19 pandemic and due to the large burden placed on hospitals, it was clear that healthcare associated SARS-CoV-2 transmission was a cause for concern for both patients and HCWs^10,11^. The three main causes for HCW infections in hospitals are HCW-to-HCW contacts, HCW-to-Patient contacts or environmental contamination^12–16^. Those different, and often interleaved, transmission routes, can complexity the viral circulation tracing in our hospitals. Transmission clusters are a way to obtain useful data, along with robust epidemiological investigations, for a better understanding of viral circulation and adequate prevention measures. Several studies have shown that combined genomic and epidemiological investigations can provide valuable information for the detection of transmission events inside hospital wards^17–20^. However, investigating clusters through genomic and phylogenetic analysis alone appears strongly limited due to SARS-CoV-2 low genetic diversity^4^. Thus, the need for complementary extensive epidemiological data is crucial and the use of phylogenetic data for such analysis is debated.

Here, we proposed to study the course of the COVID-19 epidemic in the two earliest involved major hospitals of the Paris urban area, using sequencing and phylogenetic analysis of SARS-CoV-2 complete genomes from 736 patients and healthcare-workers (HCWs), along the year of 2020. Furthermore, using phylogenetic clustering and epidemiological data from these two geographically close hospitals, we studied possible SARS-CoV-2 clusters between HCWs and patients inside BCB and PSL hospitals.

## Materials and methods

### Ethical statement

The study was carried out in accordance with the Declaration of Helsinki. This work was a retrospective non-interventional study with no addition to standard care procedures. Reclassification of biological remnants into research material after completion of the ordered virological tests and all experimental protocols were approved by the local interventional review board of Bichat-Claude-Bernard and Pitié-Salpêtrière hospitals. According to the French Public Health Code (CSP Article L.1121-1.1) such protocols are exempted from individual informed consent.

### Participants

This retrospective study was carried out in two French hospitals located in Paris urban area, Bichat Claude-Bernard hospital (BCB) and Pitié-Salpêtrière hospital (PSL). We included patients and health care workers (HCWs) diagnosed with COVID-19 from January to December 2020 with a RT-PCR SARS-CoV-2 Cycle threshold (Ct) of 30 or less, except for 8 patients with Ct > 30 successfully amplified and sequenced before the introduction of the 30 Ct threshold for sequencing. Patients and HCWs characteristics were collected from medical records and included gender, age, trip abroad in the 15 days prior to COVID-19 diagnosis, contact with a positive case of COVID-19, hospitalization and intensive care unit (ICU) requirement, hospitalization duration and death.

### Full genome sequencing

Complete genome sequencing was performed using either Illumina RNA Prep with Enrichment (Ref: 20040537, Illumina) and the Respiratory Virus Oligos Panel V2 (ref: 20044311, Illumina®) on NovaSeq (2 × 100 bp) or the Artic V.3 protocol on Minion Oxford Nanopore devices. For both procedure, RNase free PCR-grade water was used as negative controls. Consensus sequences were generated with local bioinformatics tools: mapping with Bowtie v.2.3.4.3^21^ against NC_045512.2 for reads obtained on an illumina platform or using Minimap2 v.2.15^22^ for the alignment on the reference genome and BCFtools v.1.10.2^23^ for the variant calling. Two different thresholds were used to consider a position rightly called, (i) if less than 10 reads gave the same nucleotide as the reference or if less than 30 gave a single nucleotide variant, the position was called as N, (ii) viruses sequenced with a median depth of less than 200 reads per positions and viruses with more than 15% (4500) of undetermined positions were discarded (Supplementary Table 1). The 736 consensus sequences have been deposited on GISAID database. Nextstrain^24^ and PANGO lineages^25^ SARS-CoV-2 classifications were used to assign a clade and lineage to each strain, respectively.

### Phylogenetic and clustering analysis

After genome selections, 736 reconstructed full SARS-CoV-2 genomes were aligned using mafft v7.450^26^ with 2932 worldwide sequences extracted from GISAID database and subsampled proportionally to the number of reported infections per country per week along 2020. The sequences were aligned one by one to the Wuhan Hu-1 genome (NC_045512.2) with the --keeplength option to keep numbering of positions consistent. The resulting alignment was then fed to IQTREE v2.0^27^ with a GTR + G nucleotide substitution model, 1000 ultrafast bootstrap replicates and using Wuhan Hu-1 as an outgroup to produce an accurate maximum likelihood tree. Dating of the internal nodes was obtained via the rLSD2 package implementing the LSD2 algorithm^28^ with R v4.0.5. Tree-based clustering was performed using TreeCluster^29^ with the “Max clade” method and a pairwise distance threshold of 8.4e-5 substitutions per base (corresponding to 2.5 SNPs), according to the two following conditions: (a) the maximum pairwise distance between leaves in the cluster is at most t (threshold); (b) the leaves in the cluster must define a clade in the phylogenetic tree. Several pairwise SNP distance thresholds were tested prior to perform the clustering analysis and 2.5 SNPs dissimilarities appears to be the most accurate (Supplementary Table 2). Lower thresholds were too stringent to detect most of suspected clusters and higher thresholds detected many small false positive clusters due to the low genetic diversity displayed by the SARS-CoV-2 especially in early 2020. Because of the very low SARS-CoV-2 diversity, and very low bootstrap values obtained for all the tree nodes, we did not used a branch support threshold for cluster definition.

### Epidemiological data

Socio-demographic data as well as administrative data were extracted from the hospital information system (ORBIS): sampling date, date of symptoms onset, medical ward, COVID-19 status at admission, occurrence of a previous trip abroad or in another French region, date and duration of hospitalization/ICU hospitalization and date of death. Concerning HCWs, we collected information about their jobs, the nature of their jobs (medical or non-medical) and the hospital unit or administrative office where they were working.

### Statistical analysis

Continuous variables were expressed as the median and interquartile range [IQR]. Discrete variables were expressed as numbers and percentages (%). GraphPad 9.1.0 was used to perform non-parametric tests, specifically Mann–Whitney *U* tests for quantitative data, Fisher’s exact tests and Pearson’s Chi-squared tests for qualitative data.

## Results

### Participants’ characteristics

A total of 736 participants were included with a median age of 53 [34–71] years and 48% of male. Three quarters required hospitalization and 20% were admitted into ICU. Non-hospitalized participants with asymptomatic to moderate infections were essentially HCWs and outpatients attending at the COVID-19 screening units tested positive. The median duration of hospitalization was 10 [3–21] days and 21% died. Forty-two (5.7%) travelled abroad in the 15 days preceding the diagnosis and 15% reported contact with a positive case of COVID-19. Between the two hospitals, the included participants presented a few significant differences: they were younger in PSL hospital, less hospitalized and with shorter hospitalization duration (Table 1). Those differences are due to the higher proportion of HCWs within participants from PSL hospital (BCB 15.7% versus PSL 42.2%), and there was no difference in the mean age of patients from both hospitals (62 *versus* 60 years old for patients from BCB and PSL, respectively, p = 0.22). However, the hospitalization in ICU (BCB 18% *versus* PSL 22%) and the prevalence of death (BCB 22% *versus* PSL 19%) were similar within the two sites. Two hundred and seven participants (28%) were HCWs. Compared to patients, HCWs were more frequently female (72% *versus* 44%, *p* < 0.0001), were younger (34 *versus* 62 years, *p* < 0.0001) and less frequently hospitalized (22% *versus* 83%, *p* < 0.0001) (Table 1). Among the sixteen HCWs (22%) requiring hospitalization, none required ICU admission nor died of COVID-19.

**Table 1:** Characteristics of patients and health care workers included in this study and recruited in Bichat Claude-Bernard and Pitié-Salpêtrière hospitals, two French hospitals located in the Parisian urban area, France, along 2020.

	Total (n = 736)	PSL (n = 341)	BCB (n = 395)	*P*-value	Patients (n = 529)	Health care workers (n = 207)	*P*-value
Male, n (%)	355 (48)	151 (44)	204 (53)	0.0172	298 (56)	57 (28)	< 0.0001
Age, median [IQR]	53 (34–71)	49 (32–64)	56 (36–75)	0.0001	62 (45–76)	34 (27–47)	< 0.0001
Ct value, median [IQR]	21.9 (17.7–24.8)	23.7 (21.8–25.9)	18.8 (15.8–22.8)	< 0.0001	22 (17.4–25.1)	21.8 (18.4–24.1)	0.8279
Trip	42/561 (7.5)	10/166 (6)	32/395 (8)	0.4833	41/497 (8)	1/64 (2)	0.0726
Contact with a positive case	68/463 (15)	46/166 (28)	22/297 (7)	< 0.0001	61/446 (14)	7/18 (39)	0.009
Hospitalisation, n (%)	429/568 (76)	146/212 (69)	283/356 (79)	0.0048	413/495 (83)	16/73 (22)	< 0.0001
Hospitalisation ICU, n (%)	126/632 (20)	74/337 (22)	52/295 (18)	0.1948	126/472 (27)	0/160 (0)	*–*
Total hospitalization days, median [IQR]	10 (3–21)	9 (0–22)	11.5 (6–19)	0.0025	11 (4–21)	0 (0–0)	< 0.0001
ICU hospitalization days, median [IQR]	12.5 (6–23)	15 (8–25)	10 (4–19)	0.0177	–	12 (6–23.2)	–
Death, n (%)	114/553 (21)	39/206 (19)	75/347 (22)	0.5144	114/482 (24)	0/71 (0)	–
Patients, n (%)	529 (72)	197/341 (58)	332/395 (84)	< 0.0001	529 (100)	0 (0)	–

*BCB* Bichat Claude-Bernard, *n* number, *IQR* interquartile, *ICU* intensive care unit, *PSL* Pitié-Salpêtrière, – not applicable.

Statistical analyses were performed by Fisher’s exact test for qualitative data or Mann–Whitney U tests for quantitative data.

### SARS-CoV-2 molecular diversity

To confirm the robustness of our phylogenetic analysis of SARS-CoV-2 genomes, we produced a time-scaled tree and estimated a mutation rate of 3.9e−4 mutations/site/year and time of the most recent common ancestor (tMRCA) at the 2019-11-06. This estimation is consistent with our current knowledge on SARS-CoV-2 emergence and evolution.

**Figure 1:**
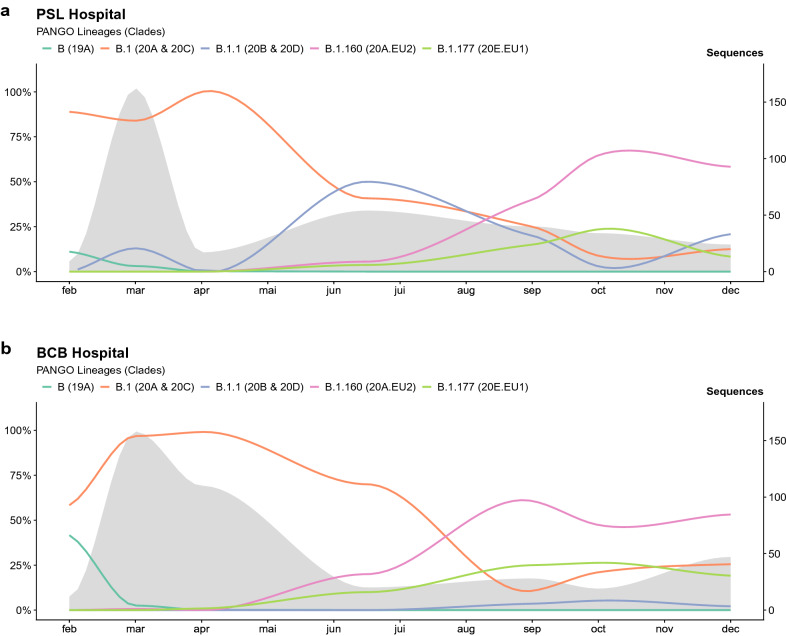
Overall distribution of PANGO lineages identified during year 2020 within Pitié-Salpêtrière hospital (**a**) and Bichat Claude-Bernard hospital (**b**), two French hospitals located in the Parisian urban area. The x-axis represents each month, the left y-axis the percentage of each lineage and the right y-axis the number of SARS-CoV-2 genomes sequenced per month within our study (grey zone).

The SARS-CoV-2 clade diversity between BCB and PSL hospitals was significantly different (*p* < 0.0001) (Fig. 1)*.* Clade 20A was more prevalent in BCB (55% *versus* 38%), whereas clades 20B and 20D were only found in PSL and represented about 8% of all strains along 2020 (Supplementary Table 3). By the end of February 2020, at the early stage of the epidemic, we found 6/22 genomes (27%) belonging to the 19A clade prevalent in Asia. Four were isolated from patients coming back from China, one from Egypt and one from a general practitioner infected by a Chinese tourist patient^30^. At that time, 20A (32%) and 20C (36%) were already the two most represented clades, characterized by the emergence of the D614G substitution in the Spike protein. During the spring outbreak (March–April), the 19A clade disappeared (0.02%) and the 20A clade became the most prevalent (63% *versus* 31% and 6% for 20C and all other clades, respectively). From May to August, we observed a decrease of both 20A (38%) and 20C (12%) clades and an increase of the 20B clade (34%). These three clades, composed of SARS-CoV-2 strains from B.1 lineage, constitute most of the COVID-19 first wave in our hospitals and stayed prevalent during the summer. Clades 20A.EU2 (lineage B.1.160) and 20E.EU1 (lineage B.1.177) emerged in late August. By the end of September, the clade 20A.EU2 became the most prevalent (47%), while clades 20E.EU1 (16%) and 20A (18%) plateaued at similar levels. From September to December 2020, these three clades represented most of the second wave viral diversity (89%) with 20A.EU2 still being the most prevalent (52%) (Fig. 1).

### Phylogenetic clustering

Tree-based phylogenetic clustering enabled us to detect 57 clusters composed of 2 to 11 participants, with a median size of 2 [2, 3]. SARS-CoV-2 genomes from 574 participants (78%) did not belong to any cluster. These clusters were included within a median time span of 6 days [1–15] with a median time interval between samples inside each cluster of 4 days [1–8]. A majority of clusters, 45/57 (79%), included viral genomes from only two to three participants and 12/57 (21%) included more than 3 participants with a median size of 4.5 participants [4–5.75], hereafter called large clusters. The proportion of HCWs was significantly higher in clusters compared to non-clusters (35% *versus* 26%, *p* = 0.03) whereas age, hospitalization (standard or ICU) and proportion of deaths were not different (Table 2). More precisely, this overrepresentation of HCWs was significant within large clusters (38% *versus* 26%, *p* = 0.04) but not within smaller clusters (32% *versus* 26%, *p* = 0.20). Clades distribution was significantly different between non-clusters and clusters (*p* < 0.0001). Indeed, the clade 20A.EU2 was less represented (9% *versus* 16%, *p* = 0.01) in clusters whereas the clade 20C was more frequent (33% *versus* 20%, *p* = 0.0004).

**Table 2:** Comparison of non-clusters and clusters sequences identified by phylogenetic clustering analysis and associated to epidemiological evidences.

	Non-clusters (n = 574)	Clusters (n = 162)	*P*-value	Small clusters (2 to 3 participants) (n = 96)	*P*-value	Large clusters (4 to 11 participants) (n = 66)	P-value	Epidemiological clusters (n = 69)	*P*-value
Male, n (%)	291 (51)	64 (40)	0.01	43 (46)	0.32	21 (32)	0.004	22 (32)	0.003
Age, median [IQR]	53 [34 – 71]	56 [36 – 75]	0.18	54 [36 – 69]	0.95	59 [37 – 83]	0.02	54 [35 – 82]	0.07
Hospitalisation, n (%)	344/454 (76)	85/114 (75)	0.80	50/70 (71)	0.45	35/44 (80)	0.71	27/40 (67)	0.25
Hospitalisation ICU, n (%)	95/490 (19)	31/142 (22)	0.55	23/85 (27)	0.11	8/57 (14)	0.37	5/62 (8)	0.03
Death, n (%)	84/441 (19)	30/112 (27)	0.08	17/67 (25)	0.25	13/45 (29)	0.12	15/40 (37)	0.01
HCWs n (%)	150 (26)	56 (35)	0.03	31 (32)	0.20	25 (38)	0.04	34 (49)	< 0.0001
19A, n (%)	11 (2)	4 (2)	0.001	4 (4)	0.02	0 (0)	< 0.0001	0 (0)	0.0007
20A, n (%)	279 (49)	68 (42)		36 (38)		32 (48)		33 (48)	
20B, n (%)	39 (7)	17 (10)		4 (4)		13 (20)		15 (22)	
20C, n (%)	113 (20)	54 (33)		33 (34)		21 (32)		13 (19)	
20D, n (%)	5 (1)	0 (0)		0 (0)		0 (0)		0 (0)	
20A.EU2, n (%)	92 (16)	14 (9)		14 (15)		0 (0)		8 (12)	
20E.EU1, n (%)	35 (6)	5 (3)		5 (5)		0 (0)		0 (0)	
Number of clusters		57		45		12		17	
Cluster size, median [IQR]		2 [2, 3]		2 [2–2]		4.5 [4–5.75]		3 [2–5]	

*N* number, *IQR* interquartile range, *ICU* intensive care unit, *HCWs* health care workers.

Statistical analyses were performed against “Non-clustering group” by Fisher’s exact test and Chi-squared test for qualitative data or Mann–Whitney U test for quantitative data.

### Epidemiological links within identified clusters

**Figure 2:**
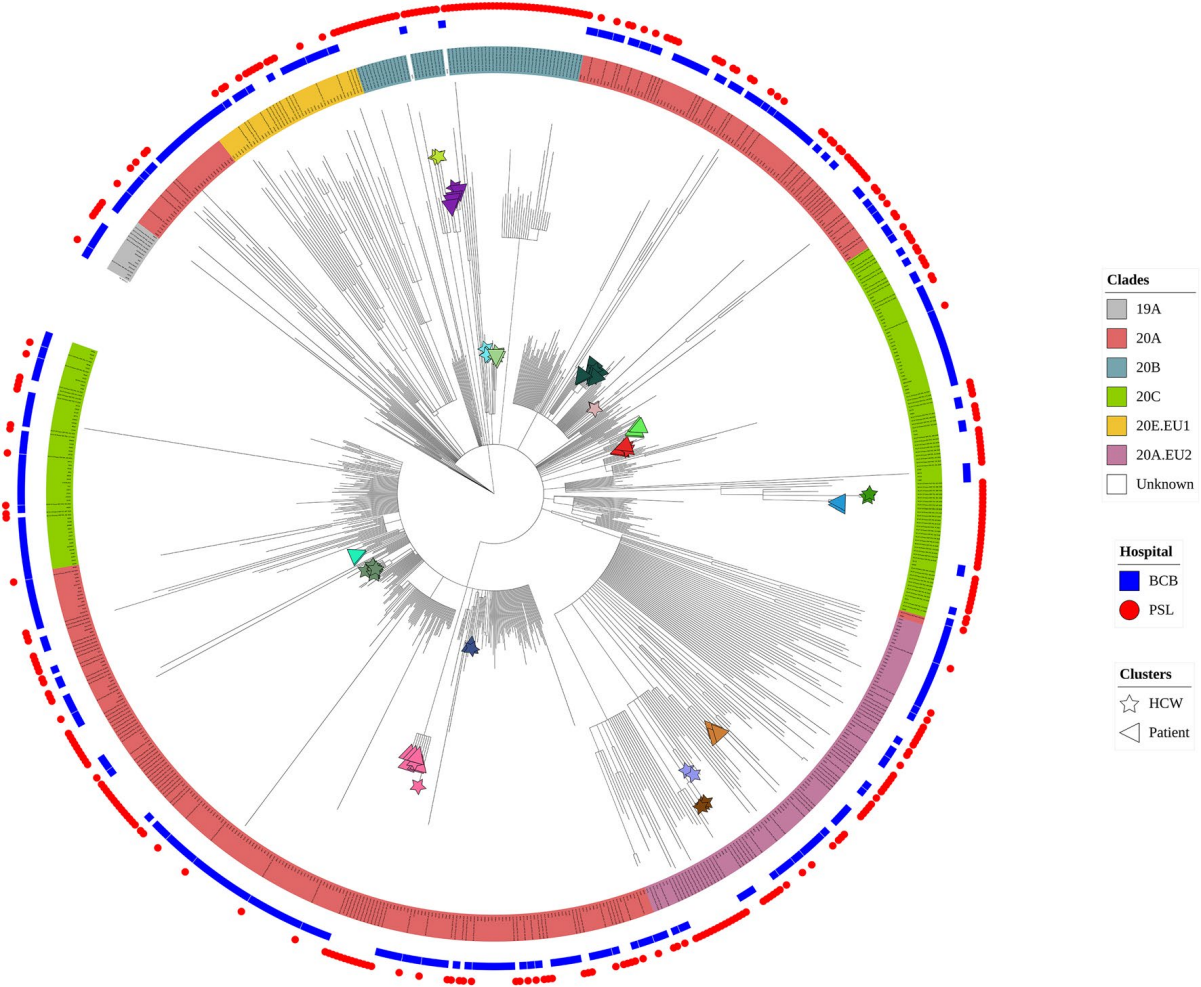
Phylogenetic tree showing 736 SARS-CoV-2 completes genomes from health care workers and patients from Bichat Claude-Bernard and Pitié-Salpêtrière hospitals, Paris, France, over the year 2020. The maximum likelihood phylogenetic tree was inferred from the alignment of the 736 reconstructed full SARS-CoV-2 genomes and 2932 worldwide sequences extracted from GISAID database, with IQTREE v2.0 with a GTR + G nucleotide substitution model, 1000 ultrafast bootstrap replicates and using Wuhan Hu-1 genome (NC_045512.2) as an outgroup. For greater clarity, 2932 worldwide representative sequences extracted from GISAID database were masked from the phylogenetic tree representation.

**Figure 3:**
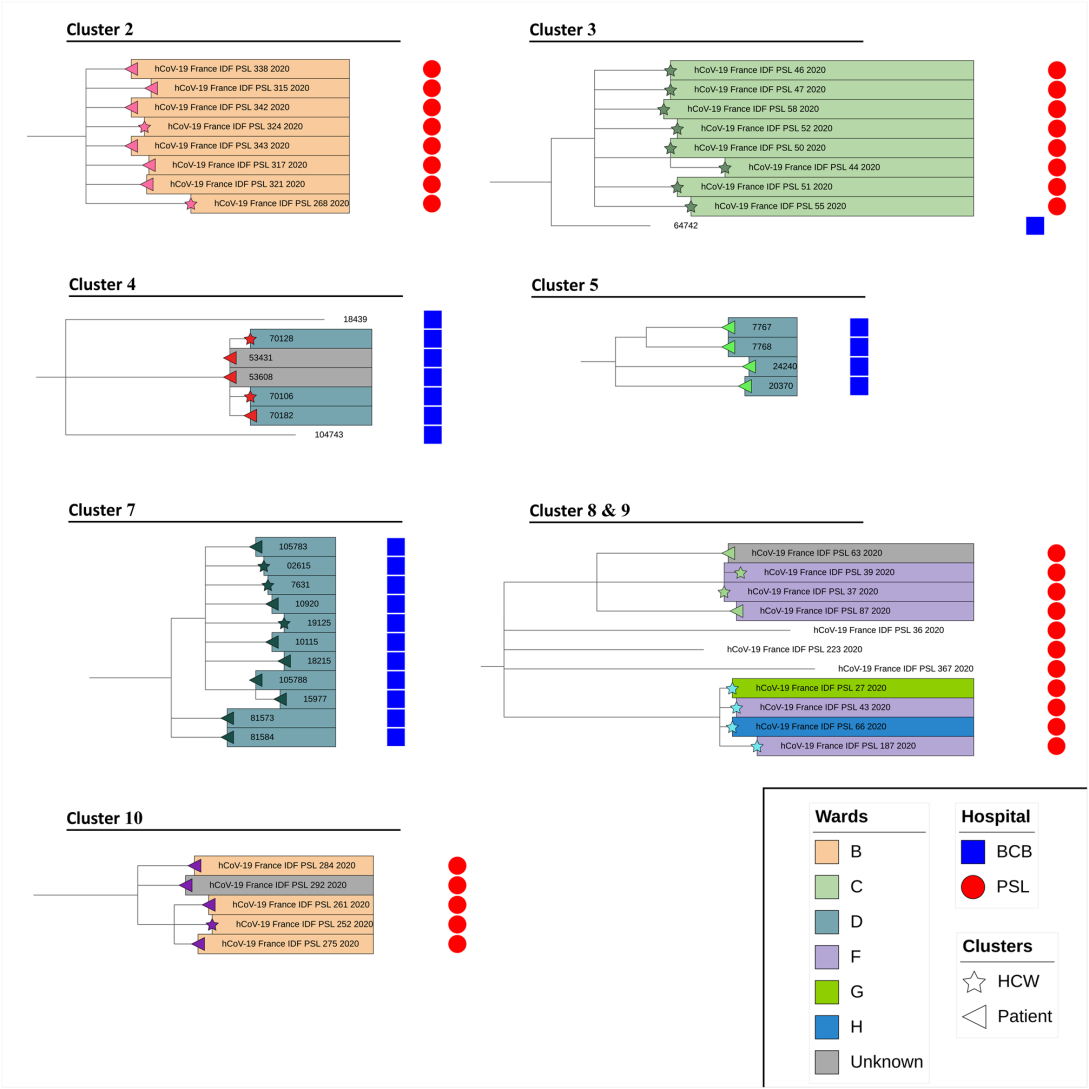
Large clusters (4 to 11 participants) identified by phylogenetic clustering and validated by epidemiological investigations. Overview of 8 large clusters from Bichat Claude-Bernard (blue squares) and Pitié-Salpêtrière (red circles) hospitals identified by phylogenetic clustering and confirmed by epidemiological investigations. Health care workers (HCWs) and patients were identified by stars or triangles symbols, respectively. Wards of participants from clusters were indicated by a color code.

Following tree-based phylogenetic clustering, we found epidemiological connections between participants (i.e. epidemiologically identified nosocomial transmissions), associated with short timespan between samples (i.e. clusters with sequences sampled within a median of 5 consecutive days [1–7]), for 17/57 (30%) clusters (Fig. 2, 3). These epidemiological clusters were significantly bigger than clusters where no connection between participants could be found (3 [2–5] *versus* 2 [2], *p* = 0.001) and had a shorter timespan (1 day [1, 2] *versus* 5 days [1–13], *p* = 0.002), when correlated to the cluster size. Furthermore, the proportion of HCWs within those epidemiological clusters were significantly higher compared to non-clusters (49% *vs* 26%, *p* < 0.0001) and to clusters with no epidemiological links (49% *vs* 24%, *p* = 0.0009). Participants from these clusters were also more susceptible to die from COVID-19 (*p* = 0.01) but less subject to ICU admission (*p* = 0.03) (Table 2). This increased mortality was notably associated with 13 patients, who died from COVID-19, from 6 clusters involving geriatric wards within our two hospitals. Overall, 6 clusters included only BCB participants and 11 only PSL participants. By definition, none of them shared sequences between the two sites. Within these 17 clusters with epidemiological links, half of included participants were HCWs; six phylogenetic clusters included both HCW and patients, seven only HCW and four clusters only patients.

Finally, one epidemiologically suspected cluster was not retrieved from the phylogenetic analysis, as the conditions required by our clustering method were not fulfilled. Nine sequences from this epidemiological outbreak, observed in a childcare center from PSL hospital in August, had a median pairwise distance of 1.8 SNPs [1.2–1.9]. However, those sequences were included in a monophyletic group together with sequences from community-acquired SARS-CoV-2 strains circulating at that time and exceeding the distance threshold we fixed, resulting in the exclusion of the cluster according to our definition. A hierarchical clustering method based on pairwise distance without consideration of any phylogenetic information allowed us to retrieve this cluster and to exclude the sequences from community acquired SARS-CoV-2. But, as a result of the very low genetic diversity displayed by the SARS-CoV-2 at the beginning of the pandemic, this method also led to the detection of several giant clusters in early 2020. Therefore, relying solely on genetic distances for clusters investigation does not seem appropriate and this highlights the usefulness of the information held by the phylogenetic tree.

## Discussion

In this study, we report SARS-CoV-2 epidemiology during the year 2020 obtained from 736 participants from two major hospitals located in Paris area, France, providing an enhanced overview of SARS-CoV-2 lineage changes in the French region with by far the largest number of infections. We also observed, with the exception of the very beginning of the pandemic presenting a very low viral diversity, a strong link between the phylogenetic clustering and epidemiologically identified clusters in each study site. This highlights the potential usefulness of phylogenetic analysis for transmission linkage and cluster investigations, but also suggests that ensuing results should be carefully interpreted within periods of low viral diversity such as the beginning of the pandemic or during or after the selective sweep of a SARS-CoV-2 variant.

At the beginning of the pandemic in the Paris area, in February 2020, the clade 19A was frequently identified with most of these cases linked with travelers and tourists coming from China. The clade 20A, associated with the emergence of the D614G mutation conferring higher infectivity and viral load^6,31^, was also present since the very beginning and quickly outpaced the clade 19A during the first wave (March to April) in France, just as in the rest of the world during the same time period^5,32^. The clade 20C, derived from the clade 20A bearing ORF3a 57H and ORF1a 265I mutations, was the second most common clade identified^32,33^. During the second part of the year 2020, global SARS-CoV-2 diversity and heterogeneity in clade composition across countries both increased. In the present study, we reported the large spread of the 20A.EU2 and 20E.EU1 clades^7,34^ in the Paris area since August. These clades emerged during the second part of the year in Europe whereas they remained anecdotal in other regions of the world^35^. Overall, the SARS-CoV-2 molecular diversity we described in the Paris urban area is in accordance with the diversity described in different European countries over 2020^36,37^. Because of the same catchment area and the shared population of patients, the molecular epidemiology between the two hospitals was similar, except for the rare clades 20B and 20D from the first wave. In PSL hospital, we reported 8% of 20B and 1% of 20D, which were not identified in the BCB hospital. This could be explained in part by the fact that 38 of the corresponding strains were included in epidemiological validated clusters from PSL hospital. Nevertheless, the highly similar diversities allowed us to check the capability of our phylogenetic reconstruction to identify specific nosocomial clusters across the two hospitals.

Beyond global epidemic surveillance, SARS-CoV-2 genome sequencing has been proposed to track and find events of local transmission, especially in high risk environments such as hospital wards^17,19,38^. However, the slow evolution rate and the quick spread of SARS-CoV-2 lineages could hinder the detection of local transmission as similar or identical strains could be found in individuals that are not closely epidemiologically linked. Indeed, previous studies have already shown that low genetic diversity of SARS-CoV-2 made the identification of clusters of transmission extremely complex^17,19^. After two years of COVID-19 spreading and underlying viral evolution, the viral diversity has increased and could obviously make the cluster detection easier. Nevertheless, in the case of a quick resurgence of viral circulation associated with the emergence of a new dominant variant, such as the Alpha^39^ or Delta^40^ variants able to quickly replace all other circulating strains, we could observed a remarkable decrease of genetic diversity. Thus, if local transmission cluster studies provide very useful information, the criteria for cluster definition should be carefully assessed in regards to the ongoing viral diversity.

Based on phylogenetic clustering, without consideration of any epidemiological data, we were able to identify 57 hypothetical clusters. Among them, 17 clusters, larger in size and spanning a shorter period, were confirmed by epidemiological investigations. Even though small false positive clusters were still detected with this method, corroborating the previously highlighted complexity to describe clusters with SARS-CoV-2, it appears to provide useful information, reinforcing the use of phylogenetic studies for nosocomial clusters investigation. However, the best parameters for the phylogenetic analysis would depend on SARS-CoV-2 molecular diversity at the time and place of the study. The choice of a genetic distance threshold to detect clusters is a balance between stringency, with the risk of missing real contamination events, and leniency, with the risk of false positive clusters. We selected a maximum genetic distance of 2.5 mutations. This distance provided meaningful results according to our investigations. Despite the geographical proximity between the two hospitals and the shared population of patients, no clusters associating participants from the two hospitals and supported by epidemiological connections could be found. However, we found 3 of such clusters by our phylogenetic reconstruction (2 clusters of 4 participants and 1 cluster of 5 participants) with participants contaminated during a close time interval and shared between the two hospitals. No links could be established to confirm an actual epidemiological connection between the participants for any of those 3 clusters. All were identified in March 2020, at the beginning of the first wave. As all those cluster mixing the two hospitals were identified during this first wave period, when viral diversity was at its lowest, we could hypothesize that those clusters are explained by a higher probability of nearly identical strains. Nevertheless, we could also explain them by the possibility of inter-hospital transmission linked with exchange of patients or HCWs during this period, even if no such events have been strongly identified.

Our study has some limitations. Firstly, we couldn’t obtain samples from all patients and HCWs during the study period due to the absence of systematic testing. Moreover, we were not able to sequence all genomes from all positive collected samples. Thus, we may have missed cases possibly connected to a cluster. Secondly, we focused mainly on phylogenic reconstruction ability to detect clusters. Epidemiological connections between participants were essentially supported by the date of the first positive test and the ward location within the two hospitals sites. As interactions between HCWs themselves as well as with patients are very complex, precise contact tracing investigations are also necessary to apprehend the whole dynamic of those interactions and transmission networks.

In conclusion, we provide an enhanced overview of phylogenetic changes and various viral lineages coexistence over the whole year 2020 in the Paris area, one of the regions with the highest incidence in France, with the spread of the 20A.EU2 and 20E.EU1 clades since August just before the start of the French 2nd wave. We also evaluated the clustering patterns among patients and HCWs of the two earliest Parisian hospitals involved in the COVID-19 response. Small clusters may not reflect local transmission as they included 2 to 3 participants with dissimilar sampling times, and were mostly sampled at the beginning of the pandemic when viral diversity was low. However, large clusters appeared highly informative. Indeed, two-third of the large clusters were epidemiologically confirmed. More systematic phylogenetic analysis of SARS-CoV-2 strains obtained from HCWs and patients, along with comprehensive epidemiological data, should help to obtain a better view of viral transmission within our hospitals.

## Supplementary Information

Supplementary Tables.
