# Supplementary material for: Nosocomial transmission clusters and lineage diversity characterized by SARS-CoV-2 genomes from two large hospitals in Paris, France, in 2020
Source: Sci Rep. 2022 Jan 20;12:1094. doi: 10.1038/s41598-022-05085-2 (PMC8776803; doi:10.1038/s41598-022-05085-2)
Supplement: Supplementary file 1 — Supplementary Tables. [file 41598_2022_5085_MOESM1_ESM.docx]

**Title: Nosocomial transmission clusters and lineage diversity characterized by SARS-CoV-2 genomes from two large hospitals in Paris, France, in 2020**

**Valentin Leducq^1^*, Aude Jary^1^*, Antoine Bridier-Nahmias^2^, Lena Daniel^2^, Karen Zafilaza^1^, Florence Damond^2,3^, Valérie Goldstein^4^, Audrey Duval^2^, François Blanquart^5^, Vincent Calvez^1^, Diane Descamps^2,3^, Anne-Geneviève Marcelin^1^*, Benoit Visseaux^2,3^***

**Affiliations**

**^1^** Sorbonne Université, INSERM, Institut Pierre Louis d’Epidémiologie et de Santé Publique (iPLESP), AP-HP, Hôpital Pitié-Salpêtrière, Service de Virologie, Paris, FR

**^2^** Université de Paris, Inserm, UMR1137, IAME, Paris, FR

**^3^** Université de Paris, Inserm, UMR1137, IAME, Service de Virologie, Hôpital Bichat-Claude Bernard, AP-HP, Paris FR

**^4^** AP-HP, Sorbonne Université, Hôpital Pitié-Salpêtrière Charles-Foix, Service de Bactériologie Hygiène, Paris, FR

**^5^** Centre for Interdisciplinary Research in Biology (CIRB), Collège de France, CNRS, INSERM, PSL Research University, Paris, France

**^*^** These authors contributed equally

**Correspondence** and requests for materials should be addressed to V.L.

Hôpital Pitié-Salpêtrière, Service de Virologie, 47-83 Bd de l'hôpital 75013 Paris, FRANCE. Tel: +33142177558, email: valentin.leducq@sorbonne-universite.fr

**Supplementary Table 1:** Ct value and undetermined position (N) count and percentage for each 736 SARS-CoV-2 genomes. A result was considered as negative if above 40 Ct, as recommended by the manufacturers of the assays used for diagnosis at the time in our hospitals. For some patients, tested with the FilmArray (BioFire, BioMérieux) assay, Ct values are not available as no Ct is provided by this qualitative assay.

| Sequence ID | Ct value | N | % N |
| --- | --- | --- | --- |
| 80108 | NA | 4200 | 14,0% |
| 54727 | 30,3 | 3537 | 11,8% |
| 81059 | 24,8 | 2420 | 8,1% |
| 28423 | 15,8 | 2334 | 7,8% |
| 577 | 14,5 | 2261 | 7,6% |
| 79441 | 21,5 | 2217 | 7,4% |
| 68278 | 23,2 | 2209 | 7,4% |
| 74083 | 22,9 | 2118 | 7,1% |
| 30392 | 15,8 | 2098 | 7,0% |
| 38117 | 23,4 | 2098 | 7,0% |
| 15112 | 18,5 | 2096 | 7,0% |
| 7243 | 16,1 | 2038 | 6,8% |
| 21500 | 18,5 | 1998 | 6,7% |
| 24328 | 24,3 | 1935 | 6,5% |
| 30541 | 24,3 | 1804 | 6,0% |
| 10774 | 12,5 | 1738 | 5,8% |
| 33415 | 24,7 | 1738 | 5,8% |
| 3678 | 16,8 | 1720 | 5,8% |
| hCoV-19_France_IDF_PSL_178_2020 | 27,0 | 1679 | 5,6% |
| 32931 | 35,4 | 1678 | 5,6% |
| 9148 | 15,0 | 1656 | 5,5% |
| 72150 | 22,6 | 1553 | 5,2% |
| 5305 | 17,6 | 1388 | 4,6% |
| 19987 | 22,3 | 1375 | 4,6% |
| 5461 | 17,5 | 1344 | 4,5% |
| 69781 | 24,7 | 1273 | 4,3% |
| 12137 | 21,1 | 1224 | 4,1% |
| 27588 | 25,7 | 1217 | 4,1% |
| 105752 | 28,2 | 1216 | 4,1% |
| 101220 | 22,2 | 1184 | 4,0% |
| 96499 | 26,8 | 1168 | 3,9% |
| 5341 | 25,5 | 1163 | 3,9% |
| 22341 | 36,4 | 1162 | 3,9% |
| hCoV-19_France_IDF_PSL_232_2020 | 29,7 | 1161 | 3,9% |
| 70092 | 26,8 | 1159 | 3,9% |
| 85463 | 22,6 | 1149 | 3,8% |
| 69763 | 19,6 | 1067 | 3,6% |
| HURJC105432 | 29,0 | 1021 | 3,4% |
| 81043 | 26,2 | 990 | 3,3% |
| 12139 | 20,5 | 974 | 3,3% |
| 81110 | 20,2 | 973 | 3,3% |
| 114369 | 34,0 | 967 | 3,2% |
| 7839 | 10,7 | 961 | 3,2% |
| 14489 | 18,4 | 940 | 3,1% |
| 50662 | 27,1 | 901 | 3,0% |
| VOR | NA | 897 | 3,0% |
| 19989 | 12,9 | 889 | 3,0% |
| 50997 | 28,0 | 886 | 3,0% |
| 70675 | 26,1 | 878 | 2,9% |
| 96956 | 18,9 | 878 | 2,9% |
| 105664 | 22,7 | 873 | 2,9% |
| 105788 | 29,0 | 871 | 2,9% |
| 105836 | 26,6 | 869 | 2,9% |
| 105762 | 21,4 | 868 | 2,9% |
| 105683 | 25,5 | 864 | 2,9% |
| 99765 | 25,2 | 862 | 2,9% |
| 96434 | 20,7 | 858 | 2,9% |
| hCoV-19_France_IDF_PSL_497_2020 | 25,6 | 855 | 2,9% |
| 105715 | 25,3 | 852 | 2,8% |
| 102458 | 24,3 | 840 | 2,8% |
| 5785 | NA | 834 | 2,8% |
| 105659 | 14,5 | 833 | 2,8% |
| 104713 | 14,9 | 819 | 2,7% |
| 83549 | 19,0 | 814 | 2,7% |
| 96621 | 19,6 | 806 | 2,7% |
| 64742 | 15,5 | 788 | 2,6% |
| 83361 | 16,0 | 777 | 2,6% |
| 104666 | 16,6 | 777 | 2,6% |
| 105713 | 23,8 | 775 | 2,6% |
| 32529 | 21,0 | 758 | 2,5% |
| hCoV-19_France_IDF_PSL_206_2020 | 27,9 | 754 | 2,5% |
| 70897 | 20,6 | 748 | 2,5% |
| 51156 | 24,3 | 746 | 2,5% |
| hCoV-19_France_IDF_PSL_237_2020 | 29,8 | 745 | 2,5% |
| 16965 | 16,2 | 742 | 2,5% |
| 65566 | 23,3 | 734 | 2,5% |
| 104638 | 14,7 | 724 | 2,4% |
| 70128 | 21,6 | 681 | 2,3% |
| 105898 | 24,0 | 674 | 2,3% |
| 101187 | 20,2 | 648 | 2,2% |
| 81568 | 15,0 | 645 | 2,2% |
| 98398 | 20,6 | 644 | 2,2% |
| 101298 | 17,9 | 643 | 2,2% |
| 101113 | 19,9 | 641 | 2,1% |
| hCoV-19_France_IDF_PSL_493_2020 | 25,5 | 639 | 2,1% |
| 96917 | 18,9 | 624 | 2,1% |
| 871091 | 27,0 | 619 | 2,1% |
| 96432 | 17,3 | 614 | 2,1% |
| 96467 | 24,7 | 613 | 2,0% |
| 53237 | 13,8 | 610 | 2,0% |
| 109961 | 26,4 | 609 | 2,0% |
| 80511 | 17,6 | 596 | 2,0% |
| hCoV-19_France_IDF_PSL_181_2020 | 27,1 | 594 | 2,0% |
| 109959 | 17,7 | 580 | 1,9% |
| 19660 | 24,7 | 572 | 1,9% |
| 48292 | 27,3 | 559 | 1,9% |
| 20817 | 22,2 | 555 | 1,9% |
| 19945 | 16,6 | 551 | 1,8% |
| 101792 | 19,1 | 549 | 1,8% |
| 50503 | 19,8 | 545 | 1,8% |
| 48923 | 23,4 | 539 | 1,8% |
| 53181 | 16,8 | 532 | 1,8% |
| 81106 | 24,1 | 531 | 1,8% |
| 28599 | NA | 530 | 1,8% |
| 89608 | 29,9 | 526 | 1,8% |
| 98419 | 25,0 | 523 | 1,7% |
| 70440 | 15,4 | 513 | 1,7% |
| 105919 | NA | 513 | 1,7% |
| 32789 | 21,0 | 511 | 1,7% |
| 104743 | 17,8 | 511 | 1,7% |
| 7631 | 23,3 | 508 | 1,7% |
| hCoV-19_France_IDF_PSL_278_2020 | 17,3 | 505 | 1,7% |
| 11492 | 23,2 | 504 | 1,7% |
| 52433 | 25,8 | 503 | 1,7% |
| 57738 | 31,9 | 502 | 1,7% |
| 64728 | 19,2 | 501 | 1,7% |
| 33995 | 20,8 | 500 | 1,7% |
| 20438 | 23,4 | 498 | 1,7% |
| 28293 | 24,2 | 497 | 1,7% |
| 35364 | 25,0 | 494 | 1,7% |
| 69737 | 17,6 | 491 | 1,6% |
| 3666 | 28,7 | 490 | 1,6% |
| 63217 | 22,4 | 490 | 1,6% |
| 15451 | 19,8 | 487 | 1,6% |
| 101232 | 15,1 | 486 | 1,6% |
| 100990 | 28,4 | 480 | 1,6% |
| 109839 | 14,1 | 476 | 1,6% |
| 109900 | 21,3 | 474 | 1,6% |
| 665 | 18,4 | 473 | 1,6% |
| 99743 | 17,6 | 472 | 1,6% |
| 50333 | 17,4 | 464 | 1,6% |
| 109834 | 15,3 | 464 | 1,6% |
| 925548 | 15,0 | 464 | 1,6% |
| 110001 | 22,4 | 462 | 1,5% |
| 69170 | 14,4 | 457 | 1,5% |
| hCoV-19_France_IDF_PSL_222_2020 | 29,0 | 455 | 1,5% |
| 7662 | 26,3 | 450 | 1,5% |
| 109999 | 19,3 | 450 | 1,5% |
| 113937 | 19,8 | 449 | 1,5% |
| TAL25720 | 17,0 | 435 | 1,5% |
| 102391 | 20,3 | 430 | 1,4% |
| 70416 | 14,9 | 426 | 1,4% |
| 96529 | 16,4 | 395 | 1,3% |
| 106817 | 18,0 | 391 | 1,3% |
| 4269 | 19,4 | 389 | 1,3% |
| 98500 | 17,3 | 389 | 1,3% |
| 18703 | 27,2 | 388 | 1,3% |
| hCoV-19_France_IDF_PSL_229_2020 | 29,5 | 371 | 1,2% |
| 7995 | 10,5 | 357 | 1,2% |
| SHIYu89879 | 20,0 | 349 | 1,2% |
| GRAEm2655 | 32,0 | 342 | 1,1% |
| hCoV-19_France_IDF_PSL_236_2020 | 29,8 | 341 | 1,1% |
| 107145 | 20,2 | 337 | 1,1% |
| 88594 | 23,1 | 335 | 1,1% |
| 46955 | 9,7 | 323 | 1,1% |
| 6459 | 26,4 | 318 | 1,1% |
| 96879 | 17,8 | 318 | 1,1% |
| 81584 | 18,7 | 307 | 1,0% |
| 53602 | 20,6 | 304 | 1,0% |
| 97473 | 17,0 | 304 | 1,0% |
| hCoV-19_France_IDF_PSL_394_2020 | 24,7 | 304 | 1,0% |
| 2628 | 25,7 | 302 | 1,0% |
| 7914 | 13,4 | 302 | 1,0% |
| 53267 | 14,8 | 302 | 1,0% |
| 63212 | 16,9 | 302 | 1,0% |
| 64638 | 19,7 | 302 | 1,0% |
| 70141 | 20,8 | 301 | 1,0% |
| 12671 | 19,4 | 300 | 1,0% |
| 1247 | 24,1 | 299 | 1,0% |
| 64601 | 13,4 | 299 | 1,0% |
| 65776 | 16,9 | 299 | 1,0% |
| 489 | 17,8 | 298 | 1,0% |
| 13043 | 17,9 | 298 | 1,0% |
| 85464 | 21,8 | 298 | 1,0% |
| 30565 | 20,8 | 297 | 1,0% |
| 31271 | 19,1 | 296 | 1,0% |
| 96373 | 13,6 | 296 | 1,0% |
| 53431 | 17,8 | 295 | 1,0% |
| 53402 | 19,5 | 292 | 1,0% |
| 64511 | 27,1 | 290 | 1,0% |
| 20124 | 26,2 | 289 | 1,0% |
| 111725 | 23,5 | 288 | 1,0% |
| 50226 | 19,7 | 287 | 1,0% |
| 25425 | 24,8 | 286 | 1,0% |
| 53405 | 16,9 | 286 | 1,0% |
| 63570 | 15,2 | 285 | 1,0% |
| 101417 | 21,5 | 285 | 1,0% |
| 25143 | 28,1 | 284 | 0,9% |
| 49418 | 25,3 | 284 | 0,9% |
| 53326 | 23,2 | 284 | 0,9% |
| 96491 | 19,6 | 284 | 0,9% |
| 109846 | 14,3 | 284 | 0,9% |
| TOU36779 | 22,0 | 284 | 0,9% |
| 50469 | 15,0 | 283 | 0,9% |
| 53649 | 12,6 | 283 | 0,9% |
| 7508 | 25,9 | 282 | 0,9% |
| 96446 | 12,3 | 282 | 0,9% |
| 38997 | 10,6 | 281 | 0,9% |
| 40999 | 19,7 | 281 | 0,9% |
| 677 | 15,9 | 280 | 0,9% |
| 64497 | 22,8 | 279 | 0,9% |
| ZHAL106076 | 16,0 | 278 | 0,9% |
| 48351 | 21,3 | 277 | 0,9% |
| 61252 | 17,8 | 277 | 0,9% |
| 50431 | 16,4 | 276 | 0,9% |
| 53185 | 20,4 | 276 | 0,9% |
| 53415 | 17,8 | 276 | 0,9% |
| 120729 | 15,9 | 276 | 0,9% |
| 50264 | 19,9 | 275 | 0,9% |
| 75991 | 23,4 | 275 | 0,9% |
| hCoV-19_France_IDF_PSL_234_2020 | 29,7 | 274 | 0,9% |
| 16754 | 20,3 | 273 | 0,9% |
| 50136 | 17,7 | 273 | 0,9% |
| 53608 | 17,8 | 273 | 0,9% |
| 62807 | 14,1 | 273 | 0,9% |
| 70106 | 17,6 | 272 | 0,9% |
| 33571 | 15,2 | 271 | 0,9% |
| GAL96647 | 25,1 | 270 | 0,9% |
| hCoV-19_France_IDF_PSL_315_2020 | 21,9 | 270 | 0,9% |
| 52315 | 17,7 | 269 | 0,9% |
| 70111 | 16,5 | 269 | 0,9% |
| 109841 | 14,7 | 269 | 0,9% |
| 21044 | 15,5 | 268 | 0,9% |
| 25249 | 15,9 | 268 | 0,9% |
| 38361 | 20,2 | 268 | 0,9% |
| 40897 | 29,9 | 267 | 0,9% |
| 65861 | 25,9 | 266 | 0,9% |
| 941032 | 15,0 | 266 | 0,9% |
| 67174 | 14,3 | 265 | 0,9% |
| 105783 | 16,7 | 265 | 0,9% |
| 51149 | 19,0 | 264 | 0,9% |
| 69960 | 15,2 | 264 | 0,9% |
| 46683 | 17,6 | 263 | 0,9% |
| 70161 | 16,2 | 262 | 0,9% |
| 32965 | 23,7 | 260 | 0,9% |
| 49715 | 20,9 | 260 | 0,9% |
| 915196 | 22,0 | 260 | 0,9% |
| ESP105086 | 26,9 | 260 | 0,9% |
| 24319 | 28,8 | 259 | 0,9% |
| 36508 | 22,0 | 259 | 0,9% |
| 3591 | 18,2 | 258 | 0,9% |
| 73976 | 16,6 | 258 | 0,9% |
| 917322 | 29,0 | 258 | 0,9% |
| 19598 | 19,6 | 257 | 0,9% |
| 38993 | 22,8 | 254 | 0,8% |
| 18155 | 18,8 | 252 | 0,8% |
| ZHA7147 | 30,2 | 252 | 0,8% |
| 48 | 25,3 | 250 | 0,8% |
| 19195 | 22,7 | 250 | 0,8% |
| 33482 | 17,0 | 250 | 0,8% |
| 7467 | 21,6 | 249 | 0,8% |
| 50508 | 17,0 | 249 | 0,8% |
| 893387 | 24,0 | 249 | 0,8% |
| hCoV-19_France_IDF_PSL_213_2020 | 28,3 | 247 | 0,8% |
| 48474 | 19,6 | 245 | 0,8% |
| 24299 | 15,2 | 244 | 0,8% |
| 24337 | 20,9 | 244 | 0,8% |
| 47812 | 19,8 | 244 | 0,8% |
| hCoV-19_France_IDF_PSL_264_2020 | 29,3 | 241 | 0,8% |
| 7639 | 21,2 | 240 | 0,8% |
| 8663 | 13,0 | 240 | 0,8% |
| 11478 | 15,8 | 237 | 0,8% |
| 17079 | 15,4 | 236 | 0,8% |
| 29241 | 22,7 | 231 | 0,8% |
| hCoV-19_France_IDF_PSL_208_2020 | 28,0 | 231 | 0,8% |
| 4594 | 26,8 | 230 | 0,8% |
| 92560 | 18,4 | 230 | 0,8% |
| 20436 | 13,4 | 227 | 0,8% |
| hCoV-19_France_IDF_PSL_243_2020 | 30,0 | 227 | 0,8% |
| 20370 | 12,8 | 225 | 0,8% |
| 70182 | 17,3 | 223 | 0,7% |
| hCoV-19_France_IDF_PSL_273_2020 | 21,9 | 220 | 0,7% |
| 105875 | 16,4 | 215 | 0,7% |
| 99742 | 24,5 | 213 | 0,7% |
| hCoV-19_France_IDF_PSL_224_2020 | 29,0 | 212 | 0,7% |
| 3856 | 12,6 | 210 | 0,7% |
| 15454 | 15,6 | 207 | 0,7% |
| 56317 | 21,1 | 204 | 0,7% |
| 49802 | 25,6 | 200 | 0,7% |
| 19140 | 17,7 | 195 | 0,7% |
| 101242 | 15,5 | 195 | 0,7% |
| 919065 | 25,0 | 194 | 0,6% |
| hCoV-19_France_IDF_PSL_565_2020 | 23,8 | 182 | 0,6% |
| hCoV-19_France_IDF_PSL_593_2020 | 30,3 | 180 | 0,6% |
| hCoV-19_France_IDF_PSL_502_2020 | 26,3 | 179 | 0,6% |
| 3663 | 26,9 | 174 | 0,6% |
| 40921 | 23,5 | 171 | 0,6% |
| 78129 | 15,9 | 165 | 0,6% |
| 68129 | 25,9 | 161 | 0,5% |
| 62139 | 15,3 | 157 | 0,5% |
| 49772 | 17,1 | 136 | 0,5% |
| 3559 | 20,1 | 135 | 0,5% |
| hCoV-19_France_IDF_PSL_328_2020 | 30,0 | 114 | 0,4% |
| hCoV-19_France_IDF_PSL_165_2020 | 26,5 | 108 | 0,4% |
| hCoV-19_France_IDF_PSL_129_2020 | 25,3 | 107 | 0,4% |
| hCoV-19_France_IDF_PSL_144_2020 | 25,4 | 107 | 0,4% |
| 3675 | 21,0 | 105 | 0,4% |
| 28307 | 17,4 | 99 | 0,3% |
| hCoV-19_France_IDF_PSL_190_2020 | 27,5 | 95 | 0,3% |
| 19343 | 14,9 | 93 | 0,3% |
| hCoV-19_France_IDF_PSL_701_2020 | 29,6 | 88 | 0,3% |
| 15933 | 18,7 | 83 | 0,3% |
| hCoV-19_France_IDF_PSL_226_2020 | 29,1 | 80 | 0,3% |
| hCoV-19_France_IDF_PSL_321_2020 | 30,5 | 79 | 0,3% |
| hCoV-19_France_IDF_PSL_217_2020 | 28,6 | 77 | 0,3% |
| 99790 | 20,0 | 76 | 0,3% |
| 68542 | 22,0 | 74 | 0,2% |
| 8228 | 17,0 | 71 | 0,2% |
| hCoV-19_France_IDF_PSL_204_2020 | 27,9 | 71 | 0,2% |
| KER107630 | 28,6 | 71 | 0,2% |
| hCoV-19_France_IDF_PSL_196_2020 | 27,6 | 70 | 0,2% |
| hCoV-19_France_IDF_PSL_338_2020 | 18,7 | 70 | 0,2% |
| hCoV-19_France_IDF_PSL_510_2020 | 24,6 | 70 | 0,2% |
| hCoV-19_France_IDF_PSL_317_2020 | 16,9 | 69 | 0,2% |
| 15798 | 18,2 | 68 | 0,2% |
| 16892 | 19,4 | 68 | 0,2% |
| 20420 | 25,0 | 68 | 0,2% |
| hCoV-19_France_IDF_PSL_292_2020 | 21,3 | 68 | 0,2% |
| hCoV-19_France_IDF_PSL_501_2020 | 27,0 | 68 | 0,2% |
| hCoV-19_France_IDF_PSL_185_2020 | 27,2 | 67 | 0,2% |
| 36505 | 16,0 | 66 | 0,2% |
| hCoV-19_France_IDF_PSL_127_2020 | 25,2 | 66 | 0,2% |
| 63182 | 14,5 | 65 | 0,2% |
| 4606 | 27,2 | 64 | 0,2% |
| 8786 | 25,3 | 64 | 0,2% |
| hCoV-19_France_IDF_PSL_210_2020 | 28,2 | 64 | 0,2% |
| hCoV-19_France_IDF_PSL_275_2020 | 16,6 | 64 | 0,2% |
| 18373 | 14,0 | 63 | 0,2% |
| hCoV-19_France_IDF_PSL_279_2020 | 21,0 | 63 | 0,2% |
| hCoV-19_France_IDF_PSL_280_2020 | 21,2 | 63 | 0,2% |
| 63677 | 15,2 | 62 | 0,2% |
| hCoV-19_France_IDF_PSL_131_2020 | 25,4 | 62 | 0,2% |
| hCoV-19_France_IDF_PSL_284_2020 | 19,3 | 62 | 0,2% |
| 19648 | 17,2 | 61 | 0,2% |
| hCoV-19_France_IDF_PSL_205_2020 | 27,9 | 61 | 0,2% |
| hCoV-19_France_IDF_PSL_300_2020 | 21,3 | 61 | 0,2% |
| 6376 | 25,9 | 60 | 0,2% |
| 47702 | 18,0 | 60 | 0,2% |
| hCoV-19_France_IDF_PSL_268_2020 | 23,6 | 60 | 0,2% |
| hCoV-19_France_IDF_PSL_283_2020 | 28,3 | 60 | 0,2% |
| 10920 | 21,1 | 59 | 0,2% |
| hCoV-19_France_IDF_PSL_186_2020 | 27,2 | 59 | 0,2% |
| 7059 | 22,2 | 58 | 0,2% |
| 11270 | 14,0 | 58 | 0,2% |
| 32995 | 17,0 | 58 | 0,2% |
| hCoV-19_France_IDF_PSL_141_2020 | 25,8 | 58 | 0,2% |
| hCoV-19_France_IDF_PSL_154_2020 | 26,3 | 58 | 0,2% |
| hCoV-19_France_IDF_PSL_194_2020 | 27,6 | 58 | 0,2% |
| hCoV-19_France_IDF_PSL_257_2020 | 28,1 | 58 | 0,2% |
| 2120 | 25,8 | 57 | 0,2% |
| 2745 | 20,1 | 57 | 0,2% |
| 24240 | 13,8 | 57 | 0,2% |
| FAC900 | 14,6 | 57 | 0,2% |
| TOU7077 | 20,5 | 57 | 0,2% |
| 7768 | 15,8 | 56 | 0,2% |
| 10296 | 19,4 | 56 | 0,2% |
| 101075 | 14,2 | 56 | 0,2% |
| hCoV-19_France_IDF_PSL_197_2020 | 27,6 | 56 | 0,2% |
| hCoV-19_France_IDF_PSL_228_2020 | 29,3 | 56 | 0,2% |
| hCoV-19_France_IDF_PSL_252_2020 | 20,6 | 56 | 0,2% |
| hCoV-19_France_IDF_PSL_458_2020 | 27,9 | 56 | 0,2% |
| RIM104461 | 24,0 | 56 | 0,2% |
| 19491 | 18,0 | 55 | 0,2% |
| 20123 | 18,2 | 55 | 0,2% |
| 96915 | 18,4 | 55 | 0,2% |
| hCoV-19_France_IDF_PSL_172_2020 | 26,7 | 55 | 0,2% |
| hCoV-19_France_IDF_PSL_506_2020 | 27,7 | 55 | 0,2% |
| 2615 | 19,8 | 54 | 0,2% |
| 12128 | 22,0 | 54 | 0,2% |
| 52456 | 16,7 | 54 | 0,2% |
| 89308 | 10,3 | 54 | 0,2% |
| hCoV-19_France_IDF_PSL_134_2020 | 25,5 | 54 | 0,2% |
| hCoV-19_France_IDF_PSL_150_2020 | 26,1 | 54 | 0,2% |
| hCoV-19_France_IDF_PSL_151_2020 | 26,2 | 54 | 0,2% |
| hCoV-19_France_IDF_PSL_343_2020 | 22,5 | 54 | 0,2% |
| 6523 | 17,6 | 53 | 0,2% |
| 18211 | 25,2 | 53 | 0,2% |
| 20421 | 14,6 | 53 | 0,2% |
| 96454 | 17,3 | 53 | 0,2% |
| hCoV-19_France_IDF_PSL_324_2020 | 18,1 | 53 | 0,2% |
| hCoV-19_France_IDF_PSL_471_2020 | 25,0 | 53 | 0,2% |
| 3571 | 14,8 | 52 | 0,2% |
| 19545 | 20,5 | 52 | 0,2% |
| 56321 | 18,9 | 52 | 0,2% |
| 917318 | 15,0 | 52 | 0,2% |
| GRA107124 | 25,8 | 52 | 0,2% |
| hCoV-19_France_IDF_PSL_288_2020 | 14,6 | 52 | 0,2% |
| hCoV-19_France_IDF_PSL_291_2020 | 19,5 | 52 | 0,2% |
| hCoV-19_France_IDF_PSL_342_2020 | 20,3 | 52 | 0,2% |
| 2164 | 19,9 | 51 | 0,2% |
| 8261 | 12,8 | 51 | 0,2% |
| 11281 | 13,2 | 51 | 0,2% |
| 70172 | 16,0 | 51 | 0,2% |
| 81573 | 14,9 | 51 | 0,2% |
| hCoV-19_France_IDF_PSL_133_2020 | 25,5 | 51 | 0,2% |
| hCoV-19_France_IDF_PSL_168_2020 | 26,6 | 51 | 0,2% |
| hCoV-19_France_IDF_PSL_256_2020 | 19,0 | 51 | 0,2% |
| MRA310465 | 19,1 | 51 | 0,2% |
| SHI | NA | 51 | 0,2% |
| 12438 | 23,0 | 50 | 0,2% |
| 25398 | 24,2 | 50 | 0,2% |
| 53269 | 18,8 | 50 | 0,2% |
| hCoV-19_France_IDF_PSL_350_2020 | 24,0 | 50 | 0,2% |
| 50441 | 16,9 | 49 | 0,2% |
| hCoV-19_France_IDF_PSL_142_2020 | 25,8 | 49 | 0,2% |
| hCoV-19_France_IDF_PSL_281_2020 | 23,6 | 49 | 0,2% |
| 19125 | 21,3 | 48 | 0,2% |
| 24471 | 15,8 | 48 | 0,2% |
| 52326 | 18,0 | 48 | 0,2% |
| 942536 | 19,0 | 48 | 0,2% |
| hCoV-19_France_IDF_PSL_367_2020 | 26,1 | 48 | 0,2% |
| CAU012921 | 28,7 | 47 | 0,2% |
| hCoV-19_France_IDF_PSL_230_2020 | 29,5 | 47 | 0,2% |
| 14751 | 15,0 | 46 | 0,2% |
| hCoV-19_France_IDF_PSL_166_2020 | 26,5 | 46 | 0,2% |
| hCoV-19_France_IDF_PSL_375_2020 | 26,8 | 46 | 0,2% |
| 29250 | 14,1 | 45 | 0,2% |
| 121490 | 13,6 | 45 | 0,2% |
| hCoV-19_France_IDF_PSL_135_2020 | 25,5 | 45 | 0,2% |
| hCoV-19_France_IDF_PSL_235_2020 | 29,8 | 45 | 0,2% |
| hCoV-19_France_IDF_PSL_381_2020 | 21,2 | 45 | 0,2% |
| hCoV-19_France_IDF_PSL_393_2020 | 22,9 | 45 | 0,2% |
| hCoV-19_France_IDF_PSL_415_2020 | 22,3 | 45 | 0,2% |
| hCoV-19_France_IDF_PSL_416_2020 | 20,0 | 45 | 0,2% |
| hCoV-19_France_IDF_PSL_420_2020 | 25,8 | 45 | 0,2% |
| hCoV-19_France_IDF_PSL_424_2020 | 25,6 | 45 | 0,2% |
| hCoV-19_France_IDF_PSL_451_2020 | 26,2 | 45 | 0,2% |
| hCoV-19_France_IDF_PSL_462_2020 | 26,6 | 45 | 0,2% |
| hCoV-19_France_IDF_PSL_466_2020 | 25,2 | 45 | 0,2% |
| hCoV-19_France_IDF_PSL_567_2020 | 23,1 | 45 | 0,2% |
| hCoV-19_France_IDF_PSL_578_2020 | 25,9 | 45 | 0,2% |
| 7637 | 16,9 | 44 | 0,1% |
| 21053 | 16,7 | 44 | 0,1% |
| hCoV-19_France_IDF_PSL_138_2020 | 25,7 | 44 | 0,1% |
| hCoV-19_France_IDF_PSL_202_2020 | 27,9 | 44 | 0,1% |
| hCoV-19_France_IDF_PSL_253_2020 | 16,9 | 44 | 0,1% |
| hCoV-19_France_IDF_PSL_422_2020 | 25,9 | 44 | 0,1% |
| hCoV-19_France_IDF_PSL_436_2020 | 25,7 | 44 | 0,1% |
| hCoV-19_France_IDF_PSL_600_2020 | 25,9 | 44 | 0,1% |
| 29244 | 16,9 | 43 | 0,1% |
| hCoV-19_France_IDF_PSL_459_2020 | 24,5 | 43 | 0,1% |
| 20106 | 14,8 | 42 | 0,1% |
| 66174 | 15,7 | 42 | 0,1% |
| 109962 | 17,0 | 42 | 0,1% |
| hCoV-19_France_IDF_PSL_271_2020 | 21,5 | 42 | 0,1% |
| hCoV-19_France_IDF_PSL_431_2020 | 20,8 | 42 | 0,1% |
| 19416 | 17,3 | 41 | 0,1% |
| hCoV-19_France_IDF_PSL_149_2020 | 26,1 | 41 | 0,1% |
| hCoV-19_France_IDF_PSL_261_2020 | 17,5 | 41 | 0,1% |
| hCoV-19_France_IDF_PSL_433_2020 | 21,7 | 41 | 0,1% |
| hCoV-19_France_IDF_PSL_564_2020 | 23,0 | 41 | 0,1% |
| 52387 | 21,0 | 40 | 0,1% |
| 85918 | 11,3 | 40 | 0,1% |
| hCoV-19_France_IDF_PSL_368_2020 | 25,3 | 40 | 0,1% |
| hCoV-19_France_IDF_PSL_369_2020 | 26,8 | 40 | 0,1% |
| hCoV-19_France_IDF_PSL_384_2020 | 21,1 | 40 | 0,1% |
| hCoV-19_France_IDF_PSL_432_2020 | 24,7 | 40 | 0,1% |
| hCoV-19_France_IDF_PSL_435_2020 | 24,3 | 40 | 0,1% |
| hCoV-19_France_IDF_PSL_464_2020 | 21,0 | 40 | 0,1% |
| hCoV-19_France_IDF_PSL_475_2020 | 26,2 | 40 | 0,1% |
| 109068 | 19,8 | 39 | 0,1% |
| hCoV-19_France_IDF_PSL_123_2020 | 25,1 | 39 | 0,1% |
| hCoV-19_France_IDF_PSL_130_2020 | 23,9 | 39 | 0,1% |
| hCoV-19_France_IDF_PSL_143_2020 | 25,9 | 39 | 0,1% |
| hCoV-19_France_IDF_PSL_195_2020 | 27,6 | 39 | 0,1% |
| hCoV-19_France_IDF_PSL_263_2020 | 23,8 | 39 | 0,1% |
| hCoV-19_France_IDF_PSL_286_2020 | 25,3 | 39 | 0,1% |
| hCoV-19_France_IDF_PSL_365_2020 | 23,9 | 39 | 0,1% |
| hCoV-19_France_IDF_PSL_395_2020 | 21,3 | 39 | 0,1% |
| hCoV-19_France_IDF_PSL_397_2020 | 25,1 | 39 | 0,1% |
| hCoV-19_France_IDF_PSL_408_2020 | 21,3 | 39 | 0,1% |
| hCoV-19_France_IDF_PSL_411_2020 | 21,3 | 39 | 0,1% |
| hCoV-19_France_IDF_PSL_423_2020 | 21,8 | 39 | 0,1% |
| hCoV-19_France_IDF_PSL_434_2020 | 24,5 | 39 | 0,1% |
| hCoV-19_France_IDF_PSL_460_2020 | 21,9 | 39 | 0,1% |
| hCoV-19_France_IDF_PSL_480_2020 | 26,8 | 39 | 0,1% |
| hCoV-19_France_IDF_PSL_486_2020 | 23,7 | 39 | 0,1% |
| 10115 | 22,8 | 38 | 0,1% |
| 19537 | 17,4 | 38 | 0,1% |
| 36514 | 17,0 | 38 | 0,1% |
| hCoV-19_France_IDF_PSL_285_2020 | 26,8 | 38 | 0,1% |
| hCoV-19_France_IDF_PSL_441_2020 | 18,3 | 38 | 0,1% |
| hCoV-19_France_IDF_PSL_722_2020 | 24,6 | 38 | 0,1% |
| 3849 | 14,8 | 37 | 0,1% |
| 7767 | 14,9 | 37 | 0,1% |
| 18439 | 23,2 | 37 | 0,1% |
| hCoV-19_France_IDF_PSL_377_2020 | 25,8 | 37 | 0,1% |
| hCoV-19_France_IDF_PSL_380_2020 | 26,3 | 37 | 0,1% |
| hCoV-19_France_IDF_PSL_383_2020 | 23,5 | 37 | 0,1% |
| hCoV-19_France_IDF_PSL_386_2020 | 20,6 | 37 | 0,1% |
| hCoV-19_France_IDF_PSL_456_2020 | 22,7 | 37 | 0,1% |
| hCoV-19_France_IDF_PSL_468_2020 | 21,6 | 37 | 0,1% |
| hCoV-19_France_IDF_PSL_477_2020 | 23,8 | 37 | 0,1% |
| hCoV-19_France_IDF_PSL_488_2020 | 21,8 | 37 | 0,1% |
| hCoV-19_France_IDF_PSL_604_2020 | 24,4 | 37 | 0,1% |
| 53833 | 15,8 | 36 | 0,1% |
| 101123 | 16,7 | 36 | 0,1% |
| hCoV-19_France_IDF_PSL_274_2020 | 25,0 | 36 | 0,1% |
| hCoV-19_France_IDF_PSL_399_2020 | 20,8 | 36 | 0,1% |
| hCoV-19_France_IDF_PSL_489_2020 | 24,2 | 36 | 0,1% |
| 19418 | 13,8 | 35 | 0,1% |
| 48347 | 18,8 | 35 | 0,1% |
| 894074 | 21,0 | 35 | 0,1% |
| 7476 | 12,8 | 34 | 0,1% |
| BAY8027 | 22,8 | 34 | 0,1% |
| hCoV-19_France_IDF_PSL_199_2020 | 27,7 | 34 | 0,1% |
| hCoV-19_France_IDF_PSL_372_2020 | 18,8 | 34 | 0,1% |
| hCoV-19_France_IDF_PSL_405_2020 | 22,7 | 34 | 0,1% |
| hCoV-19_France_IDF_PSL_412_2020 | 19,5 | 34 | 0,1% |
| 692 | 18,4 | 33 | 0,1% |
| 3838 | 16,5 | 33 | 0,1% |
| 7501 | 11,4 | 33 | 0,1% |
| 15977 | 15,6 | 33 | 0,1% |
| 32532 | 13,0 | 33 | 0,1% |
| 101119 | 17,0 | 33 | 0,1% |
| 111301 | 27,0 | 33 | 0,1% |
| 943616 | 15,0 | 33 | 0,1% |
| hCoV-19_France_IDF_PSL_225_2020 | 29,1 | 33 | 0,1% |
| hCoV-19_France_IDF_PSL_388_2020 | 18,4 | 33 | 0,1% |
| hCoV-19_France_IDF_PSL_453_2020 | 21,2 | 33 | 0,1% |
| hCoV-19_France_IDF_PSL_463_2020 | 23,9 | 33 | 0,1% |
| hCoV-19_France_IDF_PSL_503_2020 | 24,9 | 33 | 0,1% |
| hCoV-19_France_IDF_PSL_545_2020 | 22,6 | 33 | 0,1% |
| hCoV-19_France_IDF_PSL_547_2020 | 22,7 | 33 | 0,1% |
| hCoV-19_France_IDF_PSL_591_2020 | 23,1 | 33 | 0,1% |
| 7457 | 14,8 | 32 | 0,1% |
| 109955 | 17,1 | 32 | 0,1% |
| hCoV-19_France_IDF_PSL_177_2020 | 26,9 | 32 | 0,1% |
| hCoV-19_France_IDF_PSL_363_2020 | 23,7 | 32 | 0,1% |
| hCoV-19_France_IDF_PSL_413_2020 | 21,5 | 32 | 0,1% |
| hCoV-19_France_IDF_PSL_417_2020 | 19,9 | 32 | 0,1% |
| hCoV-19_France_IDF_PSL_430_2020 | 19,5 | 32 | 0,1% |
| 3851 | 13,9 | 31 | 0,1% |
| 17660 | 15,4 | 31 | 0,1% |
| 18515 | 20,6 | 31 | 0,1% |
| 19477 | 13,8 | 31 | 0,1% |
| 919069 | 15,0 | 31 | 0,1% |
| hCoV-19_France_IDF_PSL_487_2020 | 22,2 | 31 | 0,1% |
| SYL85806 | 19,6 | 31 | 0,1% |
| 64713 | 13,2 | 30 | 0,1% |
| 863761 | 19,0 | 30 | 0,1% |
| hCoV-19_France_IDF_PSL_504_2020 | 24,8 | 30 | 0,1% |
| hCoV-19_France_IDF_PSL_728_2020 | 23,3 | 30 | 0,1% |
| 8488 | 15,2 | 29 | 0,1% |
| 18215 | 17,8 | 29 | 0,1% |
| 117935 | 15,4 | 29 | 0,1% |
| 919057 | 18,0 | 29 | 0,1% |
| hCoV-19_France_IDF_PSL_211_2020 | 28,3 | 29 | 0,1% |
| hCoV-19_France_IDF_PSL_223_2020 | 29,0 | 29 | 0,1% |
| 63443 | 15,6 | 28 | 0,1% |
| hCoV-19_France_IDF_PSL_364_2020 | 24,2 | 28 | 0,1% |
| hCoV-19_France_IDF_PSL_379_2020 | 19,0 | 28 | 0,1% |
| hCoV-19_France_IDF_PSL_387_2020 | 25,0 | 28 | 0,1% |
| hCoV-19_France_IDF_PSL_454_2020 | 23,0 | 28 | 0,1% |
| hCoV-19_France_IDF_PSL_597_2020 | 22,3 | 28 | 0,1% |
| hCoV-19_France_IDF_PSL_703_2020 | 22,3 | 28 | 0,1% |
| hCoV-19_France_IDF_PSL_137_2020 | 25,7 | 27 | 0,1% |
| hCoV-19_France_IDF_PSL_200_2020 | 27,8 | 27 | 0,1% |
| hCoV-19_France_IDF_PSL_414_2020 | 19,3 | 27 | 0,1% |
| hCoV-19_France_IDF_PSL_541_2020 | 23,2 | 27 | 0,1% |
| hCoV-19_France_IDF_PSL_598_2020 | 23,6 | 27 | 0,1% |
| 69753 | 15,5 | 26 | 0,1% |
| hCoV-19_France_IDF_PSL_400_2020 | 22,4 | 26 | 0,1% |
| hCoV-19_France_IDF_PSL_145_2020 | 25,9 | 25 | 0,1% |
| hCoV-19_France_IDF_PSL_146_2020 | 25,9 | 25 | 0,1% |
| hCoV-19_France_IDF_PSL_153_2020 | 26,3 | 25 | 0,1% |
| hCoV-19_France_IDF_PSL_218_2020 | 27,3 | 25 | 0,1% |
| hCoV-19_France_IDF_PSL_392_2020 | 18,7 | 25 | 0,1% |
| hCoV-19_France_IDF_PSL_410_2020 | 21,2 | 25 | 0,1% |
| hCoV-19_France_IDF_PSL_448_2020 | 23,5 | 25 | 0,1% |
| hCoV-19_France_IDF_PSL_473_2020 | 25,0 | 25 | 0,1% |
| hCoV-19_France_IDF_PSL_539_2020 | 24,4 | 25 | 0,1% |
| hCoV-19_France_IDF_PSL_189_2020 | 27,8 | 24 | 0,1% |
| hCoV-19_France_IDF_PSL_216_2020 | 28,5 | 24 | 0,1% |
| hCoV-19_France_IDF_PSL_182_2020 | 27,1 | 23 | 0,1% |
| hCoV-19_France_IDF_PSL_214_2020 | 28,4 | 23 | 0,1% |
| hCoV-19_France_IDF_PSL_605_2020 | 27,0 | 23 | 0,1% |
| hCoV-19_France_IDF_PSL_371_2020 | 27,2 | 22 | 0,1% |
| hCoV-19_France_IDF_PSL_396_2020 | 22,0 | 22 | 0,1% |
| hCoV-19_France_IDF_PSL_406_2020 | 19,3 | 22 | 0,1% |
| hCoV-19_France_IDF_PSL_559_2020 | 22,2 | 22 | 0,1% |
| 50428 | 12,8 | 21 | 0,1% |
| 52302 | 14,2 | 21 | 0,1% |
| hCoV-19_France_IDF_PSL_378_2020 | 23,3 | 21 | 0,1% |
| hCoV-19_France_IDF_PSL_470_2020 | 25,0 | 21 | 0,1% |
| hCoV-19_France_IDF_PSL_478_2020 | 26,0 | 21 | 0,1% |
| hCoV-19_France_IDF_PSL_407_2020 | 22,6 | 20 | 0,1% |
| hCoV-19_France_IDF_PSL_409_2020 | 23,4 | 20 | 0,1% |
| hCoV-19_France_IDF_PSL_469_2020 | 23,1 | 20 | 0,1% |
| hCoV-19_France_IDF_PSL_476_2020 | 24,6 | 20 | 0,1% |
| hCoV-19_France_IDF_PSL_601_2020 | 25,8 | 20 | 0,1% |
| 65016 | 14,0 | 19 | 0,1% |
| hCoV-19_France_IDF_PSL_164_2020 | 26,4 | 19 | 0,1% |
| hCoV-19_France_IDF_PSL_446_2020 | 22,4 | 19 | 0,1% |
| hCoV-19_France_IDF_PSL_538_2020 | 24,0 | 19 | 0,1% |
| hCoV-19_France_IDF_PSL_544_2020 | 19,6 | 19 | 0,1% |
| 64443 | 14,4 | 18 | 0,1% |
| hCoV-19_France_IDF_PSL_171_2020 | 26,7 | 18 | 0,1% |
| hCoV-19_France_IDF_PSL_277_2020 | 22,3 | 18 | 0,1% |
| hCoV-19_France_IDF_PSL_389_2020 | 22,8 | 18 | 0,1% |
| hCoV-19_France_IDF_PSL_461_2020 | 23,4 | 18 | 0,1% |
| 64531 | 21,5 | 17 | 0,1% |
| 100073 | 20,1 | 17 | 0,1% |
| hCoV-19_France_IDF_PSL_188_2020 | 27,3 | 17 | 0,1% |
| 50879 | 14,3 | 16 | 0,1% |
| hCoV-19_France_IDF_PSL_180_2020 | 27,0 | 16 | 0,1% |
| hCoV-19_France_IDF_PSL_390_2020 | 16,2 | 16 | 0,1% |
| hCoV-19_France_IDF_PSL_457_2020 | 22,7 | 16 | 0,1% |
| hCoV-19_France_IDF_PSL_203_2020 | 27,9 | 15 | 0,1% |
| 50544 | 13,8 | 14 | 0,0% |
| 52791 | 12,7 | 14 | 0,0% |
| 69205 | 14,5 | 14 | 0,0% |
| hCoV-19_France_IDF_PSL_479_2020 | 19,5 | 14 | 0,0% |
| hCoV-19_France_IDF_PSL_602_2020 | 21,8 | 14 | 0,0% |
| hCoV-19_France_IDF_PSL_707_2020 | 22,6 | 14 | 0,0% |
| 51161 | 14,7 | 13 | 0,0% |
| hCoV-19_France_IDF_PSL_391_2020 | 22,5 | 13 | 0,0% |
| hCoV-19_France_IDF_PSL_184_2020 | 27,2 | 12 | 0,0% |
| hCoV-19_France_IDF_PSL_167_2020 | 26,6 | 11 | 0,0% |
| hCoV-19_France_IDF_PSL_558_2020 | 22,5 | 11 | 0,0% |
| hCoV-19_France_IDF_PSL_132_2020 | 25,5 | 9 | 0,0% |
| hCoV-19_France_IDF_PSL_173_2020 | 26,7 | 9 | 0,0% |
| hCoV-19_France_IDF_PSL_40_2020 | 24,1 | 9 | 0,0% |
| hCoV-19_France_IDF_PSL_429_2020 | 21,2 | 9 | 0,0% |
| hCoV-19_France_IDF_PSL_70_2020 | 22,9 | 9 | 0,0% |
| hCoV-19_France_IDF_PSL_455_2020 | 22,6 | 8 | 0,0% |
| hCoV-19_France_IDF_PSL_187_2020 | 26,2 | 7 | 0,0% |
| hCoV-19_France_IDF_PSL_428_2020 | 22,9 | 7 | 0,0% |
| hCoV-19_France_IDF_PSL_440_2020 | 27,2 | 7 | 0,0% |
| hCoV-19_France_IDF_PSL_546_2020 | 19,1 | 7 | 0,0% |
| hCoV-19_France_IDF_PSL_553_2020 | 20,8 | 7 | 0,0% |
| hCoV-19_France_IDF_PSL_570_2020 | 21,9 | 7 | 0,0% |
| hCoV-19_France_IDF_PSL_126_2020 | 25,2 | 6 | 0,0% |
| hCoV-19_France_IDF_PSL_401_2020 | 23,0 | 6 | 0,0% |
| hCoV-19_France_IDF_PSL_500_2020 | 25,0 | 6 | 0,0% |
| hCoV-19_France_IDF_PSL_519_2020 | 21,0 | 6 | 0,0% |
| hCoV-19_France_IDF_PSL_556_2020 | 22,3 | 6 | 0,0% |
| hCoV-19_France_IDF_PSL_577_2020 | 24,4 | 6 | 0,0% |
| hCoV-19_France_IDF_PSL_465_2020 | 21,4 | 5 | 0,0% |
| hCoV-19_France_IDF_PSL_491_2020 | 23,3 | 5 | 0,0% |
| hCoV-19_France_IDF_PSL_603_2020 | 21,6 | 2 | 0,0% |
| hCoV-19_France_IDF_PSL_526_2020 | 20,5 | 1 | 0,0% |
| hCoV-19_France_IDF_PSL_572_2020 | 21,3 | 1 | 0,0% |
| hCoV-19_France_IDF_PSL_1_2020 | 14,6 | 0 | 0,0% |
| hCoV-19_France_IDF_PSL_100_2020 | 23,9 | 0 | 0,0% |
| hCoV-19_France_IDF_PSL_101_2020 | 23,9 | 0 | 0,0% |
| hCoV-19_France_IDF_PSL_102_2020 | 23,9 | 0 | 0,0% |
| hCoV-19_France_IDF_PSL_103_2020 | 23,9 | 0 | 0,0% |
| hCoV-19_France_IDF_PSL_105_2020 | 24,0 | 0 | 0,0% |
| hCoV-19_France_IDF_PSL_106_2020 | 24,0 | 0 | 0,0% |
| hCoV-19_France_IDF_PSL_107_2020 | 24,0 | 0 | 0,0% |
| hCoV-19_France_IDF_PSL_108_2020 | 24,0 | 0 | 0,0% |
| hCoV-19_France_IDF_PSL_109_2020 | 24,1 | 0 | 0,0% |
| hCoV-19_France_IDF_PSL_113_2020 | 24,4 | 0 | 0,0% |
| hCoV-19_France_IDF_PSL_114_2020 | 24,5 | 0 | 0,0% |
| hCoV-19_France_IDF_PSL_115_2020 | 24,6 | 0 | 0,0% |
| hCoV-19_France_IDF_PSL_116_2020 | 24,6 | 0 | 0,0% |
| hCoV-19_France_IDF_PSL_117_2020 | 24,6 | 0 | 0,0% |
| hCoV-19_France_IDF_PSL_118_2020 | 24,7 | 0 | 0,0% |
| hCoV-19_France_IDF_PSL_119_2020 | 24,7 | 0 | 0,0% |
| hCoV-19_France_IDF_PSL_120_2020 | 24,7 | 0 | 0,0% |
| hCoV-19_France_IDF_PSL_121_2020 | 24,7 | 0 | 0,0% |
| hCoV-19_France_IDF_PSL_14_2020 | 20,8 | 0 | 0,0% |
| hCoV-19_France_IDF_PSL_155_2020 | 22,2 | 0 | 0,0% |
| hCoV-19_France_IDF_PSL_156_2020 | 23,8 | 0 | 0,0% |
| hCoV-19_France_IDF_PSL_157_2020 | 13,7 | 0 | 0,0% |
| hCoV-19_France_IDF_PSL_160_2020 | 19,3 | 0 | 0,0% |
| hCoV-19_France_IDF_PSL_162_2020 | 18,5 | 0 | 0,0% |
| hCoV-19_France_IDF_PSL_17_2020 | 15,1 | 0 | 0,0% |
| hCoV-19_France_IDF_PSL_18_2020 | 23,8 | 0 | 0,0% |
| hCoV-19_France_IDF_PSL_19_2020 | 23,2 | 0 | 0,0% |
| hCoV-19_France_IDF_PSL_21_2020 | 22,8 | 0 | 0,0% |
| hCoV-19_France_IDF_PSL_22_2020 | 14,7 | 0 | 0,0% |
| hCoV-19_France_IDF_PSL_25_2020 | 18,7 | 0 | 0,0% |
| hCoV-19_France_IDF_PSL_26_2020 | 21,9 | 0 | 0,0% |
| hCoV-19_France_IDF_PSL_27_2020 | 22,2 | 0 | 0,0% |
| hCoV-19_France_IDF_PSL_270_2020 | 28,5 | 0 | 0,0% |
| hCoV-19_France_IDF_PSL_28_2020 | 21,8 | 0 | 0,0% |
| hCoV-19_France_IDF_PSL_29_2020 | 18,5 | 0 | 0,0% |
| hCoV-19_France_IDF_PSL_3_2020 | 18,1 | 0 | 0,0% |
| hCoV-19_France_IDF_PSL_31_2020 | 20,6 | 0 | 0,0% |
| hCoV-19_France_IDF_PSL_34_2020 | 17,5 | 0 | 0,0% |
| hCoV-19_France_IDF_PSL_36_2020 | 18,1 | 0 | 0,0% |
| hCoV-19_France_IDF_PSL_362_2020 | 23,1 | 0 | 0,0% |
| hCoV-19_France_IDF_PSL_37_2020 | 14,3 | 0 | 0,0% |
| hCoV-19_France_IDF_PSL_39_2020 | 19,0 | 0 | 0,0% |
| hCoV-19_France_IDF_PSL_43_2020 | 18,0 | 0 | 0,0% |
| hCoV-19_France_IDF_PSL_44_2020 | 22,6 | 0 | 0,0% |
| hCoV-19_France_IDF_PSL_45_2020 | 21,1 | 0 | 0,0% |
| hCoV-19_France_IDF_PSL_46_2020 | 14,1 | 0 | 0,0% |
| hCoV-19_France_IDF_PSL_47_2020 | 17,0 | 0 | 0,0% |
| hCoV-19_France_IDF_PSL_48_2020 | 17,1 | 0 | 0,0% |
| hCoV-19_France_IDF_PSL_49_2020 | 19,0 | 0 | 0,0% |
| hCoV-19_France_IDF_PSL_50_2020 | 19,1 | 0 | 0,0% |
| hCoV-19_France_IDF_PSL_51_2020 | 17,3 | 0 | 0,0% |
| hCoV-19_France_IDF_PSL_52_2020 | 18,4 | 0 | 0,0% |
| hCoV-19_France_IDF_PSL_55_2020 | 20,1 | 0 | 0,0% |
| hCoV-19_France_IDF_PSL_56_2020 | 17,6 | 0 | 0,0% |
| hCoV-19_France_IDF_PSL_58_2020 | 22,6 | 0 | 0,0% |
| hCoV-19_France_IDF_PSL_59_2020 | 22,7 | 0 | 0,0% |
| hCoV-19_France_IDF_PSL_590_2020 | 21,8 | 0 | 0,0% |
| hCoV-19_France_IDF_PSL_60_2020 | 22,8 | 0 | 0,0% |
| hCoV-19_France_IDF_PSL_61_2020 | 22,8 | 0 | 0,0% |
| hCoV-19_France_IDF_PSL_62_2020 | 22,8 | 0 | 0,0% |
| hCoV-19_France_IDF_PSL_63_2020 | 22,8 | 0 | 0,0% |
| hCoV-19_France_IDF_PSL_64_2020 | 22,8 | 0 | 0,0% |
| hCoV-19_France_IDF_PSL_65_2020 | 22,8 | 0 | 0,0% |
| hCoV-19_France_IDF_PSL_66_2020 | 22,8 | 0 | 0,0% |
| hCoV-19_France_IDF_PSL_67_2020 | 22,9 | 0 | 0,0% |
| hCoV-19_France_IDF_PSL_68_2020 | 22,9 | 0 | 0,0% |
| hCoV-19_France_IDF_PSL_7_2020 | 23,8 | 0 | 0,0% |
| hCoV-19_France_IDF_PSL_71_2020 | 20,7 | 0 | 0,0% |
| hCoV-19_France_IDF_PSL_72_2020 | 23,0 | 0 | 0,0% |
| hCoV-19_France_IDF_PSL_73_2020 | 23,0 | 0 | 0,0% |
| hCoV-19_France_IDF_PSL_74_2020 | 23,0 | 0 | 0,0% |
| hCoV-19_France_IDF_PSL_75_2020 | 23,1 | 0 | 0,0% |
| hCoV-19_France_IDF_PSL_76_2020 | 23,1 | 0 | 0,0% |
| hCoV-19_France_IDF_PSL_77_2020 | 23,1 | 0 | 0,0% |
| hCoV-19_France_IDF_PSL_79_2020 | 23,1 | 0 | 0,0% |
| hCoV-19_France_IDF_PSL_8_2020 | 24,5 | 0 | 0,0% |
| hCoV-19_France_IDF_PSL_80_2020 | 23,2 | 0 | 0,0% |
| hCoV-19_France_IDF_PSL_81_2020 | 23,2 | 0 | 0,0% |
| hCoV-19_France_IDF_PSL_82_2020 | 23,3 | 0 | 0,0% |
| hCoV-19_France_IDF_PSL_83_2020 | 23,3 | 0 | 0,0% |
| hCoV-19_France_IDF_PSL_84_2020 | 23,3 | 0 | 0,0% |
| hCoV-19_France_IDF_PSL_85_2020 | 23,3 | 0 | 0,0% |
| hCoV-19_France_IDF_PSL_86_2020 | 23,4 | 0 | 0,0% |
| hCoV-19_France_IDF_PSL_87_2020 | 23,5 | 0 | 0,0% |
| hCoV-19_France_IDF_PSL_88_2020 | 23,6 | 0 | 0,0% |
| hCoV-19_France_IDF_PSL_89_2020 | 23,6 | 0 | 0,0% |
| hCoV-19_France_IDF_PSL_90_2020 | 23,6 | 0 | 0,0% |
| hCoV-19_France_IDF_PSL_92_2020 | 23,7 | 0 | 0,0% |
| hCoV-19_France_IDF_PSL_93_2020 | 23,7 | 0 | 0,0% |
| hCoV-19_France_IDF_PSL_94_2020 | 23,8 | 0 | 0,0% |
| hCoV-19_France_IDF_PSL_95_2020 | 23,8 | 0 | 0,0% |
| hCoV-19_France_IDF_PSL_98_2020 | 23,8 | 0 | 0,0% |

**Supplementary Table 2:** Phylogenetic clustering results obtained using TreeCluster v1.0.3 with several SNP pairwise distance thresholds. Comparison between the number of small and large clusters detected according to the chosen threshold, as well as the number of clusters supported by epidemiological evidences among detected clusters.

|  | **TreeCluster SNP pairwise distance threshold** | | | | |
| --- | --- | --- | --- | --- | --- |
|  | **0 SNP** | **1 SNP** | **2 SNPs** | ***2,5 SNPs*** | **3 SNPs** |
| **Small clusters**  **2 to 3 participants (n)** | 3 | 29 | 74 | *45* | 100 |
| **Large clusters**  **4 to 11 participants (n)** | 0 | 2 | 9 | *12* | 17 |
| **Total**  **(n)** | 3 | 31 | 83 | *57* | 117 |
| **Epidemiological clusters**  **(n)** | 1 (33%) | 9 (29%) | 12 (14%) | *17 (30%)* | 17 (16%) |

*SNP: Single nucleotide polymorphism; n=number*

**Supplementary Table 3:** Distribution of the Nextstrain clades between Bichat Claude-Bernard and Pitié-Salpêtrière hospitals and between patients and health care workers.

|  | **Total (n=736)** | **PSL**  **(n=341)** | **BCB**  **(n=395)** | ***pvalue*** | **Patients**  **(n=529)** | **Helath care**  **workers (n=207)** | ***pvalue*** |
| --- | --- | --- | --- | --- | --- | --- | --- |
| **19A** | 17 (2%) | 6 (2%) | 11 (3%) | *0.4628* | 14 (3%) | 3 (1%) | *0.4220* |
| **20A** | 345 (47%) | 129 (38%) | 216 (55%) | *< 0.0001* | 268 (51%) | 77 (37%) | *0.0010* |
| **20A.EU2** | 106 (14%) | 53 (16%) | 53 (14%) | *0.4614* | 55 (10%) | 51 (25%) | *<0.0001* |
| **20B** | 56 (8%) | 56 (16%) | 0 (0%) | < *0.0001* | 31 (6%) | 25 (12%) | *0.0079* |
| **20C** | 167 (23%) | 76 (22) | 91 (23) | *0.8600* | 129 (24%) | 38 (18%) | *0.0958* |
| **20D** | 5 (1) | 5 (1%) | 0 (0%) | *0.0210* | 4 (1%) | 1 (<1%) | *>0.9999* |
| **20E.EU1** | 40 (5) | 16 (5%) | 24 (6%) | *0.4212* | 28 (5%) | 12 (6%) | *0.8566* |

*BCB: Bichat Claude-Bernard; PSL: Pitié-Salpêtrière; n=number*
